# EEG-Based Index for Timely Detecting User’s Drowsiness Occurrence in Automotive Applications

**DOI:** 10.3389/fnhum.2022.866118

**Published:** 2022-05-20

**Authors:** Gianluca Di Flumeri, Vincenzo Ronca, Andrea Giorgi, Alessia Vozzi, Pietro Aricò, Nicolina Sciaraffa, Hong Zeng, Guojun Dai, Wanzeng Kong, Fabio Babiloni, Gianluca Borghini

**Affiliations:** ^1^Laboratory of Industrial Neuroscience, Department of Molecular Medicine, Sapienza University of Rome, Rome, Italy; ^2^BrainSigns srl, Rome, Italy; ^3^Department of Anatomical, Histological, Forensic and Orthopedic Sciences, Sapienza University of Rome, Rome, Italy; ^4^School of Computer Science and Technology, Hangzhou Dianzi University, Hangzhou, China

**Keywords:** cognitive neuroscience, drowsiness, human factor, EEG, neurometrics, road safety, driving performance, neuroergonomics

## Abstract

Human errors are widely considered among the major causes of road accidents. Furthermore, it is estimated that more than 90% of vehicle crashes causing fatal and permanent injuries are directly related to mental tiredness, fatigue, and drowsiness of the drivers. In particular, driving drowsiness is recognized as a crucial aspect in the context of road safety, since drowsy drivers can suddenly lose control of the car. Moreover, the driving drowsiness episodes mostly appear suddenly without any prior behavioral evidence. The present study aimed at characterizing the onset of drowsiness in car drivers by means of a multimodal neurophysiological approach to develop a synthetic electroencephalographic (EEG)-based index, able to detect drowsy events. The study involved 19 participants in a simulated scenario structured in a sequence of driving tasks under different situations and traffic conditions. The experimental conditions were designed to induce prominent mental drowsiness in the final part. The EEG-based index, so-called *“MDrow index”*, was developed and validated to detect the driving drowsiness of the participants. The MDrow index was derived from the Global Field Power calculated in the Alpha EEG frequency band over the parietal brain sites. The results demonstrated the reliability of the proposed MDrow index in detecting the driving drowsiness experienced by the participants, resulting also more sensitive and timely sensible with respect to more conventional autonomic parameters, such as the EyeBlinks Rate and the Heart Rate Variability, and to subjective measurements (self-reports).

## Introduction

According to reports of the World Health Organization, (2021), every year 1.3 million people die as a consequence of road traffic crashes. In this context, between 20 and 50 million people suffer non-fatal injuries related to car accidents. Moreover, road traffic injuries are the leading cause of death for children and young adults aged 5–29 years and, in terms of economic weight, road traffic crashes cost most countries 3% of their gross domestic product. Among the principal causes of road traffic accidents and related mortality and non-fatal injuries are the human factors. According to Sehat et al. (2012), the human factor has a direct effect on 93% of crashes, which makes human behavior the main cause of incidents. The most common errors occurring during car driving related to human factors are strictly correlated to tiredness, mental fatigue, and drowsiness (Choi et al., 2016). In particular, drowsiness consistently increases the probability of accidents while driving. The risk of crashes in drowsy drivers is estimated to be 4–6 times higher than in awake drivers (Klauer et al., 2006). Mental drowsiness is frequently associated with a progressive decrease of reaction time, deficiency in processing of available information, errors in short-term memory and recalling, and less vigilant behavior (Ahmad Kamran et al., 2019). In the context of driving, all these aspects can lead to the loss of control of the vehicle, which later might collide with other vehicles or stationary objects, potentially amplifying the human and economic cost of the crash. Such concern is even more relevant with long-haul professional drivers, that are usually exposed to long driving sessions, possibly alternating with other physically demanding activities such as loading/unloading goods (Apostolopoulos et al., 2013). Just to provide a rough idea of the concern, an Israeli epidemiological study reported that trucks represent 6% of all vehicles, but truck crashes account for 20% of road deaths in Israel (Sabbagh-Ehrlich et al., 2005). Mental fatigue and drowsiness are univocally recognized as the main risk factors in this context (LaDou, 1988; Maycock, 1995; Häkkänen and Summala, 2000).

The automotive industry is relevantly investing in preventing drowsiness-related road accidents (Macy et al., 2014), through two main strategies: the first one consists of the development of preventive systems directly integrated into the vehicles, such as alarms and assistive modules (e.g., Lane Keeping) to support the driver when unsafe behavior is detected (Saito et al., 2016). The second one consists of the drivers’ monitoring to preventively assess the drivers’ drowsiness and, therefore, intervene before the drowsy driving behavior happens (Yeo et al., 2009; Roy et al., 2014). The first strategy foresees the support of vehicle-based sensors, i.e., interpreting the steering wheel data, steering wheel angle, the applied pressure pattern on an acceleration paddle, lane-position indicators, and pressure sensors integrated inside the car’s seat (Akhlaq et al., 2012; Reyes-Muñoz et al., 2016). The second approach is based on the analysis of physiological and neurophysiological signals collected from the driver. In fact, neurophysiological measures are considered a powerful objective way to obtain reliable information about the driver’s psychophysiological state on the basis of its mind-body relations (Borghini et al., 2014; Di Flumeri et al., 2018, 2019b; Islam et al., 2020; Marucci et al., 2021). Several previous works demonstrated how the drowsy state can be detected by the analysis of the eye-closure time, the increase of the eye-blinking frequency, monitoring of head’s movement and pose, and yawning (Vitabile et al., 2011). Other studies characterized the drivers’ drowsiness by analyzing the Electrocardiographic (ECG) signal, in particular evaluating the Heart Rate (HR) and Heart Rate Variability (HRV; Borghini et al., 2012; Ahn et al., 2016; Chowdhury et al., 2018). Besides these autonomic parameters’ analyses, a relevant number of researches on drivers’ drowsiness characterization were based on the brain cortical signals analysis (Xu et al., 2017; Yeom et al., 2017; Wang et al., 2018; Barua et al., 2019). Eoh et al. (2005) validated two Electroencephalographic (EEG)-based indexes to characterize the drowsiness, consisting respectively of Power Spectral Density (PSD)’s Alpha/Beta and PSD’s (Alpha + Theta)/Beta ratios, while performing a driving simulation task and by using an 8-channel EEG system. The increase of the PSD in low and high Alpha and Theta bands while drowsiness episodes were observed in drivers performing monotonous and poor driving tasks by Lim et al. (2014) and Lin et al. (2005). However, drowsiness generally occurs as an episodic event rather than a prolonged state (Slater, 2008), resulting in *“dozing off”* phenomena (Haworth, 2019). In this sense, other EEG studies characterized the drowsy drivers according to the Alpha spindles, which is a short (0.5–2 s) burst of high-frequency Alpha activity (Borghini et al., 2012; Lawhern et al., 2013). In this context, Simon et al. (2011) proposed an algorithm to evaluate the mental drowsiness under real car traffic conditions based on different parameters derived from the Alpha spindles, such as their peak frequency, amplitude, and duration. More recent studies (Wang et al., 2020; Cui et al., 2021) demonstrated the significant temporal correlation between the Alpha spindles and the drivers’ drowsiness episodes. A very recent review by Stancin et al. (2021) provides a detailed overview of the current state of the art from both methodological and technological perspectives, pointing out the huge variety of studies promoting different approaches and methods. Nevertheless, these studies are still far from being deployed in real applications aimed at real-time monitoring of the driver’s state. Therefore, in scientific literature there is still a lack of a synthetic index to evaluate the mental drowsiness on the basis of the driver’s brain activity, while driving and ready to be adopted in real time-like evaluation. Current in-car systems are usually based on measuring the driver’s yawning and eye blinking frequencies (Kuamr and Barwar, 2014; Deng and Wu, 2019; Liu et al., 2019). However these systems are affected by a low time resolution (minutes) to detect the phenomenon, and thus they are not effective towards episodic events. Indeed, high time resolution evaluation in such a context can play a crucial role in preventing car accidents and implementing precautionary measures in the assistive car’s modules.

The present study aimed therefore at developing and validating an innovative EEG-based monitoring technique to detect the occurrence of driving drowsiness. In particular, we aimed at:

• identifying the most prominent neurophysiological features of driver’s brain activity under drowsiness to define the EEG-based MDrow index;
• investigating the reliability of the MDrow index in detecting driving drowsiness during monotonous driving conditions;
• evaluating the capability of the MDrow index with respect to other indexes derived from autonomic signals (eye movements and ECG) in timely detecting short-term episodes of drowsiness.

## Material and Methods

### Participants

Nineteen (19) participants were recruited, on a voluntary basis, from the Sapienza University of Rome (12 males and seven females, 28.1 ± 4.7 years old) with normal or corrected-to-normal vision. The participants were selected in order to have a homogeneous sample in terms of age, driving experiences (7.89 ± 2.54 years in possession of their driving license), and cars that normally they used to drive (specifically, manual gear and not automatic one). All of them were prohibited to drink alcohol and to have heavy meals for 1 day prior to the experimental protocol, and they were asked to avoid caffeine, tea, or chocolate consumption 5 h before the experiments. Informed consent was obtained from each participant after an explanation of the study. The experiment was conducted following the principles outlined in the Declaration of Helsinki of 1975, as revised in 2008, and was approved by the Sapienza University of Rome Ethical Committee in Charge for the Department of Molecular Medicine.

### Experimental Protocol

**Figure 1:**
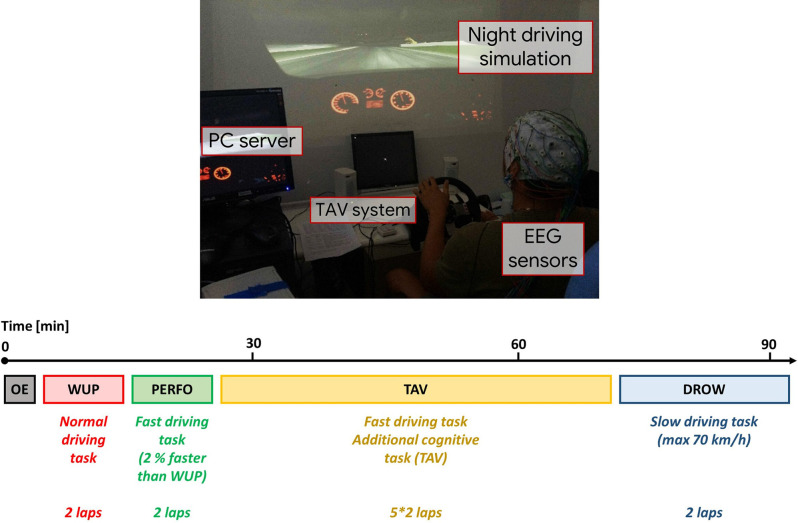
The experimental protocol consisted in driving the Alfa Romeo Giulietta QV on the Spa—Francorchamp (Belgium) track under different driving conditions. On the top, a picture of the experimental environment. On the bottom, an overview of all the experimental tasks, including the initial Open Eyes (OE) condition and all the driving tasks with related specific requests.

The experiments were performed between 2 p.m. and 5 p.m. because daytime sleepiness tends to increase during those hours (Baulk et al., 2001). The experimental main task consisted of driving a car, the Alfa Romeo—Giulietta QV (1,750 TBi, 4 cylinders, 235 HP), along the Spa—Francorchamps (Belgium) track. One experimental trial also required the participant to simultaneously perform an Alert and Vigilance Task (TAV; Kong et al., 2015). The alert stimuli, a white “X”, were presented on a monitor placed 70 (cm) from the participant just a little below the frontal direction, avoiding interference with the main screen. The vigilance stimuli were presented by two speakers placed on the left and on the right side of the driver (Figure 1).

The whole protocol was developed along 2 days. The first day of experiments was dedicated to the training of participants with the driving simulator and to the familiarization with the TAV. On the second day, the participants performed the track under the different driving conditions. Each condition consisted in driving two laps along the Spa—Francorchamps. In the first condition (warm-up, WUP) the participants did not receive any requests. Successively, the drivers were requested to perform the race reducing 2% of their total time achieved in the WUP condition (performance, PERFO). After that, the drivers had to perform the driving task by keeping the total time achieved in the PERFO condition and attending, at the same time, the TAV. Therefore, the TAV condition was equal to the PERFO condition in terms of the driving task (two laps of the circuit) but with the TAV task as a secondary task. The participants could reply to the TAV stimuli by pressing the button placed on the sides of the steering wheel. In particular, button number one (left side of the drivers) for the vigilance stimuli and button number two (right side of the drivers) for the alert stimuli. This condition aimed to enhance the task difficulty. The participants had to perform five repetitions of the TAV condition, differing in terms of cognitive demand. In particular, the cognitive demand of the TAV task was modulated by modifying the frequency of the stimuli. So the five repetitions of the TAV conditions differed in terms of stimuli rate, and they were performed in a randomized way, i.e., not from the easier (low stimuli rate) to the harder (high stimuli rate) one. The analysis of the effects related to the different TAV repetitions was out of the scope of the present study. Thus, for each analysis (see Section “Performed Analysis”) we preliminary checked if there was any significant effect between the five repetitions of the TAV condition with respect to the investigated parameters, and since no significant effects were found, we concatenated the five repetitions in a unique “TAV condition”. The last condition (DROW) was a monotonous night driving task, in which the subjects had to drive very slowly (without significantly exceeding the speed of 70 Km/h). The aim of the monotonous task after high cognitively demanding conditions was to induce boredom and finally drowsiness in the drivers. In fact, daytime with respect to circadian rhythms (in this study the early afternoon), the dark external conditions, and the low cognitive demand, especially after a fatiguing period, are normally considered the main contributing factors to the risk of drowsiness (Thiffault and Bergeron, 2003; Wang et al., 2017; Ahmad Kamran et al., 2019; Soares et al., 2020). At the end of each condition, the participant had to fill in the NASA-Task Load index (NASA-TLX; Hart and Staveland, 1988) questionnaire. The driving errors, in terms of getting off the roadway, were also noted for each participant in each experimental condition.

### Data Collection

#### EEG Signal Recording and Processing

An Electroencephalographic (EEG) signal was recorded by a digital ambulatory monitoring system (Brain Products GmbH, Germany). Sixty-one EEG channels (Fp1, Fpz, Fp2, Af7, Af3, Afz, Af4, Af8, F7, F5, F3, F1, Fz, F2, F4, F6, F8, FT7, FC5, FC3, FC1, FCz, FC2, FC4, FC6, FT8, T7, C5, C3, C1, Cz, C2, C4, C6, T8, TP7, CP5, CP3, CP1, CPz, CP2, CP4, CP6, TP8, P7, P5, P3, P1, Pz, P2, P4, P6, P8, PO7, PO3, POz, PO4, PO8, O1, Oz, and O2), placed according to the 10–10 International System, were collected simultaneously during the experiment with a sampling frequency of 250 (Hz). All the electrodes were referenced to both the earlobes, grounded to both mastoids, and the impedances were maintained around 10 (kΩ). A 50-Hz notch filter was applied to all measurements for removing main line power interference. The EEG recordings were also band-pass filtered [low-pass filter cut-off frequency: 40 (Hz), high-pass filter cut-off frequency: 2 (Hz)] and then the Independent Component Analysis (ICA) was used to remove eyeblinks and muscular artifacts. For further sources of artifacts, specific algorithms of the EEGLAB toolbox (Delorme and Makeig, 2004) were applied. Specifically, the ICA-processed signal has been then divided into 1-s-long epochs and three criteria have been applied in order to automatically recognize artefactual data. Firstly, EEG epochs with the signal amplitude exceeding ±80 μV (*Threshold* criterion) were marked as “artifacts”. Then, each EEG epoch was interpolated in order to check the slope of the trend within the considered epoch (*Trend estimation*). If such a slope is higher than 20 μV/s, the considered epoch is marked as “artifact.” Finally, the signal sample-to-sample difference (*Sample-to-sample* criterion) was analyzed: if such a difference, in terms of absolute amplitude, was higher than 25 μV, i.e., an abrupt variation (no-physiological) happened, the EEG epoch was marked as “artifact”. In the end, the EEG epochs marked as “artifacts” were removed from the EEG dataset with the aim to have a clean EEG signal to perform the analyses. In total, the 3.3% ± 2.1% (mean ± standard deviation) of EEG epochs was rejected for each participant.

From the artifact-free EEG, the Global Field Power was calculated for the EEG frequency band of interest for the mental drowsiness evaluation, which was the Alpha. The GFP was chosen as the parameter of interest describing brain EEG activity since it has the advantage of representing, in the time domain, the degree of synchronization or a specific cortical region of interest in a specific frequency band (Skrandies, 1990; Di Flumeri et al., 2016b; Cartocci et al., 2018). The Alpha band was so defined according to the Individual Alpha Frequency (IAF) value (Klimesch, 1999) computed for each participant. Since the Alpha peak is mainly prominent during rest conditions, the subjects were asked to keep their eyes open for a minute before starting the experiment. Such a condition was then used to estimate the IAF value specifically for each participant. Consequently, an EEG “strict” Alpha band was defined as Alpha = (IAF − 1) : (IAF + 1) Hz. This definition of Alpha band is more restrictive (thus “strict”) compared to the vast majority of Alpha band definitions that can be found in scientific literature, which is (IAF − 2) : (IAF + 2) Hz. This approach was selected according to Klimesch (2012), who demonstrated that a tighter band around the IAF can be considered as Alpha to avoid the impact from closer EEG frequency bands (Theta and Beta) variations on the observed phenomena in Alpha band.

The GFP was calculated over all the EEG parietal channels for each epoch using a Hanning window of the same length of the considered epoch (1 s length, that means 1 Hz of frequency resolution).

#### EOG and ECG Recordings

The electrooculographic (EOG) and electrocardiographic (ECG) signals were recorded by using the same digital ambulatory monitoring system employed for the EEG data collection. The vertical EOG pattern was estimated by analyzing the EEG Fpz channel. This analysis was based on the application of a customized version of the Reblinca method (Di Flumeri et al., 2016a) to isolate and identify the eyeblinks. The EyeBlinks Rate (EBR) parameter was estimated to perform the mental drowsiness evaluation during the different experimental conditions. The Heart Rate (HR) and Heart Rate Variability (HRV) parameters were derived from the ECG signal, collected by one electrode positioned on the Erb’s point and the two EEG references placed on the earlobes, by applying the Pan-Tompkins algorithm (Pan and Tompkins, 1985).

### Performed Analysis

#### Experimental Design Validation

As the first analysis, it was validated the experimental design. In fact, as introduced, the last driving condition (DROW) was expected to induce drowsiness in the drivers, according to scientific literature (Thiffault and Bergeron, 2003; Ahmad Kamran et al., 2019; Soares et al., 2020), since:

• the experiments were conducted after lunchtime, so the metabolism and the circadian rhythms should increase the probability of experiencing drowsiness;
• the last condition (DROW) was performed in a dark room and simulating a nighttime driving;
• the DROW condition was a monotonous and low-engaging task after a large and high demanding experimental trial.

In order to validate the last assumption, behavioral, subjective, and physiological measures were analyzed by deriving the EBR from the EOG signal and the HR and HRV from the ECG signal. The statistical analysis was performed on such physiological parameters and the subjective measurements, i.e., the NASA-TLX and the Driving Errors.

#### Neurophysiological Analysis

A preliminary statistical analysis on EEG topographic maps, a spatial representation over the scalp of a specific EEG feature, was performed in the Alpha band to identify the cortical regions related to alpha synchronization during drowsy states. In particular, for each EEG channel and along each experimental condition, the Alpha GFP was estimated. The four experimental conditions, i.e., WUP, PERFO, TAV, and DROW, were divided each in five segments of equal duration. Subsequently, for each EEG channel a Student’s *t*-test was performed between the DROW condition and, respectively, the WUP, PERFO, and TAV ones. If the test resulted was significant, the correspondent EEG channel was red-colored if the GFP increased during the DROW condition, and it was blue-colored if the GFP decreased during the DROW condition. The degree of color intensity was modulated by the *t*-value.

#### MDrow Index Development

Once identified the brain regions of interest, related to the drowsiness effect (alpha synchronization), the following procedure was adopted to define the MDrow index, in particular, the parietal brain sites were considered. All the working hypotheses of each intermediate step have been experimentally validated, as described in the following.

Based on the assumption that the Alpha rhythm reaches the maximum value in the resting state (OE condition; Klimesch, 1999), the GFPs related to all the experimental conditions were referenced to the OE condition. The Alpha-OE ratio index was therefore defined as a time-domain function:


$$Apha-OE\;\text{ratio}\;(t)=\frac{Alpha\;GFP{\left(t\right)}_{i\text{-}condition}}{\max\;\left(Alpha\;GFP_{OE\;condition}\right)}$$

where *Alpha GFP(t)_i-condition_* is the GFP computed in Alpha band in the i-th experimental condition, and the max (*Alpha GFP_OE condition_*) is the maximum value of the GFP computed in Alpha band during the OE condition.

Then, statistical analysis was performed to confirm the working hypothesis that such an index was sensitive to mental drowsiness. In particular, the distributions of the Alpha-OE ratio for each condition were compared in terms of:

- Median values to verify that this parameter was actually higher during the DROW condition, while WUP, PERFO, and TAV should not differ.
- Skewness values: according to the theory of alpha spindles (please refer to Introduction) the distribution of this parameter should show a right (i.e., positive) skewness only during the DROW condition.

Once such a hypothesis was confirmed, a threshold was determined for each participant to detect the presence of eventual peaks, i.e., index transients due to high synchronization among electrodes as a consequence of spindles. In this regard, the WUP condition was preliminarily demonstrated to be not significantly different from PERFO and TAV ones. Therefore, we used the WUP condition as a sort of “reference condition” to estimate the individual threshold as follows:


Threshold=mean (Apha-OE ratio)+3∗std (Apha-OE ratio)

Where *mean(Alpha*-*OE ratio)* is the mean value of the Alpha-OE ratio and *std(Alpha*-*OE ratio)* is its standard deviation.

Such a threshold was subsequently employed to identify the peaks of the Alpha-OE ratio along the PERFO, TAV, and DROW conditions: in particular, each local maximum of the signal exceeding the threshold was marked as a “peak”. These three conditions were so compared to verify that the number, amplitude (difference between the threshold and the maximum value of the peak), and duration (the time interval between the two local minimum points before and after a peak), of such peaks (a consequence of the alpha spindles) were higher during the DROW condition.

Finally, the convolution of the Alpha-OE ratio above the threshold with a 30-seconds-long rectangular window was estimated to integrate these three parameters in a synthetic indicator. The result of the convolution corresponded to the Mental Drowsiness (MDrow) index.

#### Statistical Analysis

All the previously mentioned comparisons have been investigated by means of statistical analyses, that were carried out using Statistica 12 software (Statsoft Europe). When performing groups analysis, for each participant, the EEG GFP in the Alpha band, EBR, HR, and HRV measured from the data collected during the OE condition were subtracted from analogous data collected during the experimental conditions in order to handle the inter-individual variability. The new EBR, HR, and HRV parameters were named respectively EBR’, HR’ and HRV’.

Firstly, the normality of the analyzed distributions was assessed by performing the Shapiro–Wilk test. If normality was confirmed, Student’s *t*-test would have been performed to pairwise compare the conditions (e.g., “DROW vs. PERFO”). In the case of non-normal distribution, the Wilcoxon signed-rank test was performed. In case of comparisons between three or more distributions, the analysis of variance (ANOVA) or its non-parametric equivalent (Friedman ANOVA) was performed. For all tests, statistical significance was set at α = 0.05.

Finally, Pearson’s repeated measure correlation (rmcorr) analysis (Bakdash and Marusich, 2017) was then used to compare the EBR’ and MDrow patterns during the condition in which the mental drowsiness was induced (DROW).

## Results

### Experimental Design Validation

**Figure 2:**
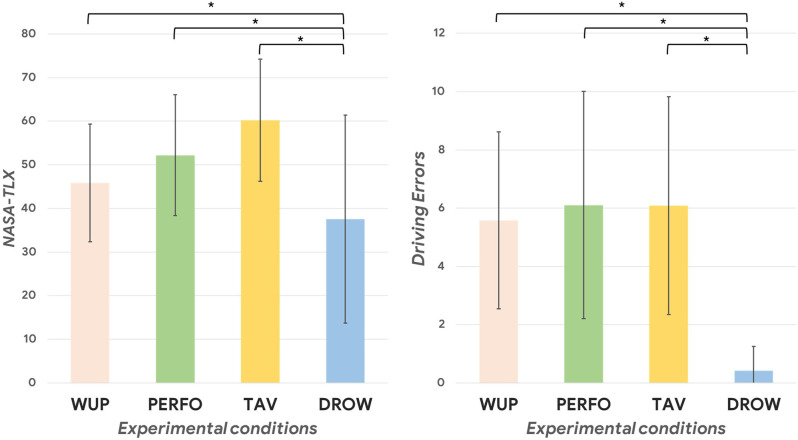
On the left, the difference in terms of perceived difficulty (mental workload score obtained from the NASA-TLX questionnaire filled at the end of each driving task) of the performed task during the different experimental conditions (all *p* < 0.01). On the right, the statistical decrease of driving errors during the DROW condition compared to the others can be observed (all *p* < 0.01). The asterisk(s) indicate whether the post-hoc paired tests are significant (*p* < 0.05).

The Friedman test performed on the NASA-TLX and the Driving Errors revealed a significant main effect among the different conditions (NASA-TLX: *p* = 0.001; Driving Errors: *p* = 0.002). Then, the Wilcoxon signed-rank test showed a significant decrease in the perceived mental workload during the DROW condition compared to the WUP, PERFO and TAV ones (DROW vs. TAV: *p* = 0.001; DROW vs. PERFO: *p* = 0.003; DROW vs. WUP: *p* = 0.01; Figure 2). Similarly, the Driving Errors parameter during the DROW condition was significantly lower compared to the others (DROW vs. TAV: *p* = 0.002; DROW vs. PERFO: *p* = 0.002; DROW vs. WUP: *p* = 0.007; Figure 2). In both analysis, no significant differences were found between WUP, PERFO, and TAV. Taken together, these results indicated that the DROW condition was perceived as simpler and monotonous by the participants with the respect to the other tasks.

The following Table 1 includes the average durations related to each driving condition and shows how the DROW condition was relevantly longer (because of the limited speed) than the others:

**Table 1:** The average durations related to each driving condition (two laps).

Driving condition	WUP	PERFO	TAV	DROW
Average time (per lap)	6’33” ± 0’54”	5’48” ± 0’51”	6’21” ± 1’08”	12’15” ± 1’47”

**Figure 3:**
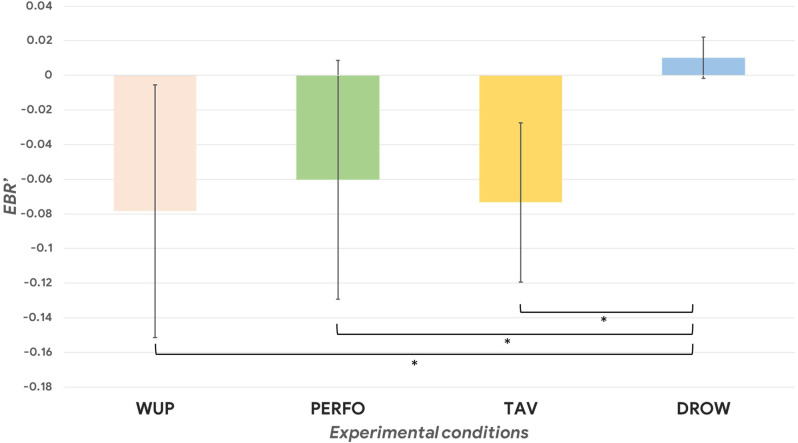
The normalized EBR (Eye Blink Rate) was significantly higher during the DROW condition compared to the WUP, PERFO, and TAV ones (*p* = 0.03). The asterisk(s) indicate whether the post-hoc paired tests are significant (*p* < 0.05).

In terms of physiological parameters, Figure 3 shows the results in terms of normalized EBR. The ANOVA performed on the EBR’, HR’ and HRV’ revealed a significant main effect among the different driving conditions (all *p* < 0.01). The *post-hoc* tests indicated that during the DROW condition the EBR’ was significantly higher compared to the others (DROW vs. TAV: *p* = 0.002; DROW vs. PERFO: *p* = 0.005; DROW vs. WUP: *p* = 0.002). Regarding the ECG analysis, both the normalized HR and HRV parameters significantly decreased during the DROW condition compared to the PERFO one (HR: *p* = 0.02; HRV: *p* = 0.01). In other words, also in terms of autonomic physiological parameters, only the DROW condition significantly differed from the others, resulting in a higher eye blink rate, a lower heart rate as well as a lower heart rate variability.

### EEG Topographic Maps

**Figure 4:**
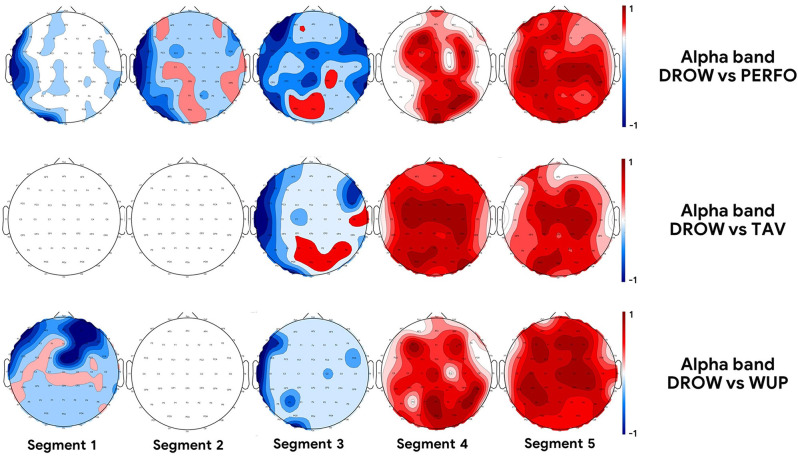
The topographic maps represent the statistical difference between the DROW condition compared to, respectively, the PERFO (first row), TAV (second row), and WUP (third row) condition in terms of power of EEG activity in Alpha band (all *p* < 0.05). Red color indicates an increase of the alpha activity, while blue indicates a decrease.

Figure 4 shows the statistical differences between the DROW condition compared respectively to the PERFO, TAV, and WUP ones (all *p* < 0.05). The analysis highlighted a common trend in terms of statistical increase of the EEG GFP in the Alpha band in frontal and parietal regions during the last two segments of the DROW condition compared to the others. In other words, WUP, PERFO, and TAV are supposed to induce a different cognitive demand depending on the specific task requests, therefore it is plausible to obtain a different “maps layout” if compared with the same condition, i.e., vs. DROW, as it happens in the first three segments. However, the similar “maps layout” arising in the last two segments means that a prominent phenomenon is appearing in the DROW task, according to our working hypothesis.

### EEG-Based Parameters Results

**Figure 5:**
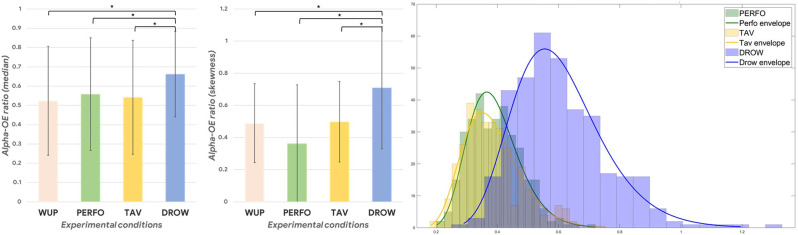
Onthe left and center, the median and the skewness of the Alpha-OE ratio index were significantly higher during the DROW condition (*p* < 0.05). On the right, the histograms of the Alpha-OE ratio distributions are represented during the PERFO, TAV, and DROW conditions. In particular, the DROW distribution is visibly larger and more skewed (i.e., there are more greater values) than the others. The asterisk(s) indicate whether the post-hoc paired tests are significant (*p* < 0.05).

The Friedman test performed on the median of the Alpha-OE ratio along each driving task highlighted a significant main effect (*p* < 0.001) among the different conditions. The Wilcoxon signed-rank test was performed to investigate any significant within effect, revealing a significant increase in the Alpha-OE ratio during the DROW condition compared to the WUP, PERFO and TAV ones (DROW vs. TAV: *p* = 0.001; DROW vs. PERFO: *p* = 0.003; DROW vs. WUP: *p* = 0.001), while no significant differences have been found among WUP, PERFO, and TAV (Figure 5). Similarly, the skewness of data distributions revealed the same pattern across the experimental conditions (DROW vs. TAV: *p* = 0.03; DROW vs. PERFO: *p* = 0.007; DROW vs. WUP: *p* = 0.02), while again no significant differences have been found among WUP, PERFO, and TAV (Figure 5). Both the presented results are resumed by the histogram represented in Figure 5.

**Figure 6:**
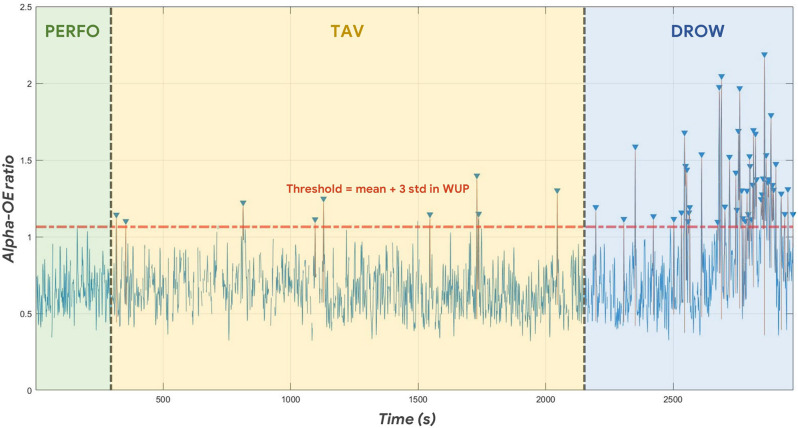
After computing the individual threshold during the WUP condition (thus not included in the following analysis), the Alpha-OE ratio peaks were identified in each experimental condition.

Once established that the WUP condition was not different in terms of Alpha-OE ratio compared to the PERFO and TAV ones, during such a condition an individual threshold was evaluated in order to identify the peaks (intended as a cue of alpha spindles) of the Alpha-OE ratio, according to the development procedure described in “MDrow Index Dvelopment” Section. Figure 6 shows the Alpha-OE ratio peaks’ identification according to the computed threshold for a representative subject.

**Figure 7:**
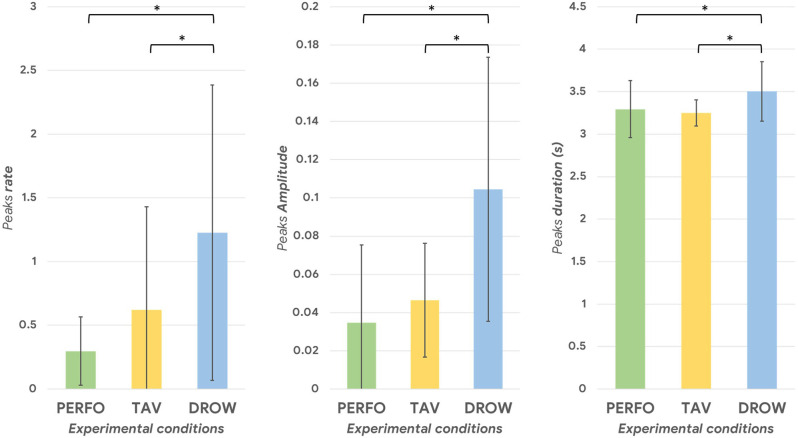
The Alpha-OE ratio peaks rate, amplitude and duration during the DROW condition were significantly higher compared to the TAV and PERFO ones (all *p* < 0.002). The asterisk(s) indicate whether the post-hoc paired tests are significant (*p* < 0.05).

The Friedman test performed on the e peaks rate, amplitude and duration indicated a significant main effect among the different driving conditions (all *p* < 0.007). The Wilcoxon signed-rank test performed on the above-mentioned parameters revealed in all the cases a significant increase during the DROW condition compared to the PERFO and TAV ones (Figure 7). The statistical results are resumed in Table 2.

**Table 2:** The statistical analysis revealed a statistical increase of the Alpha-OE peaks rate, duration and amplitude during the DROW condition (all *p* < 0.002).

*Comparison*	*Alpha-OE peaks rate*	*Alpha-OE peaks amplitude*	*Alpha-OE peaks duration*
DROW vs. TAV	*p* = 0.001	*p* = 0.0008	*p* = 0.007
DROW vs. PERFO	*p* = 0.0009	*p* = 0.0005	*p* = 0.002

### Mental Drowsiness (MDrow) Index Results

**Figure 8:**
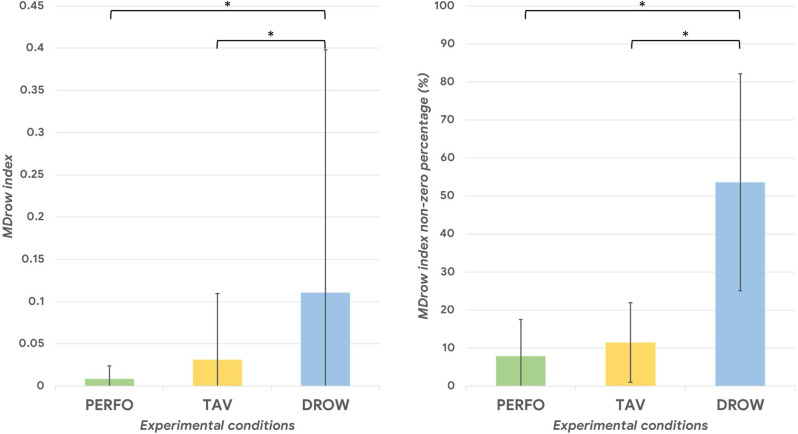
Onthe left, the MDrow index was significantly higher during the DROW condition (all *p* < 0.01). On the right, the MDrow index was higher than zero in more than 50% during the DROW condition, while its non-zero percentage was lower than 12% during the TAV and PERFO conditions. The asterisk(s) indicate whether the post-hoc paired tests are significant (*p* < 0.05).

The Friedman test performed on the MDrow index, and its non-zero percentage computed along each experimental condition, revealed a significant main effect among the different driving tasks (all *p* < 0.002). The Wilcoxon signed-rank test performed on such an index showed a significant overall increase of the index itself during the DROW condition compared to the PERFO and TAV ones (DROW vs. TAV: *p* = 0.01; DROW vs. PERFO: *p* = 0.004; Figure 8). Similarly, the non-zero percentage of the MDrow index along each experimental condition was significantly higher during the DROW condition (DROW vs. TAV: *p* = 0.001; DROW vs. PERFO: *p* = 0.0008; Figure 8).

**Figure 9:**
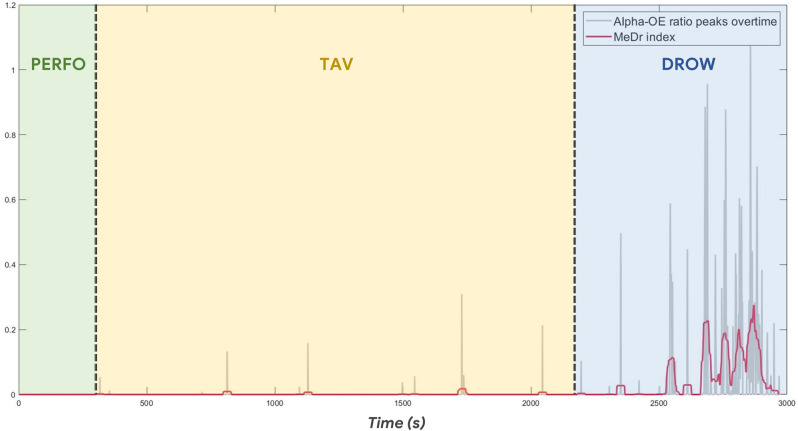
The time dynamics of the MDrow index (red bold line) along all the experimental conditions show its relationship with the GFP alpha peaks (gray background line).

Figure 9 shows the time dynamics of the MDrow index along all the experimental conditions for a representative subject.

**Figure 10:**
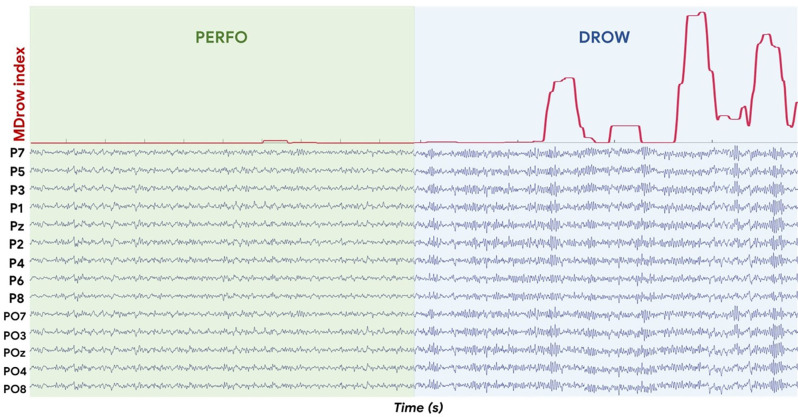
In red on the top, the MDrow index. On the bottom, the EEG signals from the parietal sites filtered in alpha band. The MDrow index is sensitive in recognizing the alpha spindles, in fact it increases while alpha spindles (i.e., synchronization phenomena) occur.

In the following Figure 10, it is provided a figure qualitatively validating the rationale behind the development of the MDrow index, i.e., the sensitivity of such an index in recognizing the alpha spindles.

### Correlations

The Pearson correlations between the EBR’ and MDrowindex performed during the DROW conditions per each participant are reported in Table 3:

**Table 3:** The Pearson correlations between the EBR’ and MDrow index performed during the DROW conditions per each participant.

Participant ID	*R* value	*p* value
Subject 1	0.3618	0.0963
Subject 2	**0.8350**	**0.0004**
Subject 3	**0.5806**	**0.0081**
Subject 4	−0.41084	0.2357
Subject 5	0.3688	0.0851
Subject 6	**0.7635**	**0.0007**
Subject 7	−0.1844	0.1863
Subject 8	**0.8381**	**0.0003**
Subject 9	**0.7697**	**0.0011**
Subject 10	**0.7017**	**0.0018**
Subject 11	**0.6993**	**0.0027**
Subject 12	−0.0819	0.4128
Subject 13	−0.0715	0.3965
Subject 14	**0.6721**	**0.0024**
Subject 15	0.3848	0.0753
Subject 16	0.3042	0.0846
Subject 17	**0.4866**	**0.0237**
Subject 18	0.2548	0.1028
Subject 19	**0.4082**	**0.0376**
**Positive and significant correlations**	**10**	

*Values in bold represent statistically significant (*p* < 0.05) correlations*.

The Pearson’s repeated measure correlation between the EBR’ and MDrow index performed during the DROW condition revealed a moderate and significant correlation (*R* = 0.49, *p* < 10^−6^).

## Discussion

### Experimental Design Validation

The impact of humans’ errors while driving in real traffic conditions can be very relevant in terms of human and economic costs (World Health Organization, 2021). Moreover, among the different human factors increasing the probability of committing errors, mental drowsiness is one of the most critical one since it immediately precedes the blow of sleepiness. The present study aimed at developing and investigating the reliability of an innovative EEG-based index for detecting the risk of mental drowsiness insurgence while driving and at comparing the sensitivity of such an index with respect to the insights provided by current physiological approaches based on eye movement and ECG derived indexes. In order to achieve these objectives, 19 participants were involved in a simulated driving protocol, divided into four tasks requiring a different level of driving performance.

The experiments were designed making the a-priori assumption that the last driving task, the DROW condition, was the one inducing mental drowsiness in the participants. In fact, according to scientific literature (Thiffault and Bergeron, 2003; Wang et al., 2017; Soares et al., 2020), the participants had to perform the study in the early afternoon after lunch (when the insurgence of drowsiness is probable), the DROW driving task was performed in dark external conditions (nighttime in the simulation task and dark room), it follows a highly demanding and fatiguing period, and it was characterized by a very low and monotonous cognitive demand.

The statistical analysis performed on the driving errors and subjective perception of the task load (NASA-TLX) validated this preliminary assumption on the experimental design: both the behavioral parameters were significantly lower (all *p* < 0.05) only during the DROW condition compared to the others. In other words, such analysis confirms that the driving task performed during the DROW condition, besides being notably longer (almost the double) than the others, was perceived as significantly low engaging compared to the others.

This evidence is confirmed by the neurophysiological brain patterns (Figure 4). More specifically, the GFP in the Alpha band was significantly higher during the DROW condition compared to the WUP, TAV, and PERFO ones (all *p* < 0.05), as expected from the scientific literature (Lim et al., 2014). While EyeBlinks Rate, Heart Rate, and its Variability highlighted the overall very low physiological activation of the participants along the DROW task. This evidence again confirms that the DROW condition induced a psychophysiological effect on the drivers compatible with drowsiness insurgence, since the EEG alpha rhythms increased, as well as the eye blinks rate, while heart rate and its variability decreased (Borghini et al., 2012).

### EEG-Based Approach for Characterizing Driving Drowsiness

Apart from validating the experimental design, neurophysiological evidence of a relevant increase of Alpha activity on both frontal and parietal sites supported our working hypothesis of deriving the MDrow index from the increased synchronization of parietal sites in terms of Alpha activity, according also to previous scientific literature (Eoh et al., 2005; Borghini et al., 2012). The GFP was so chosen as the EEG-related indicator for representing in a synthetic way the synchronous activation in the alpha band of a specific cortical area, in our case the parietal one. In particular, for each participant, such indicator computed along the tasks has been related to the maximum achieved along the OE condition, i.e., a resting state when the alpha activity is supposed to achieve the maximum individual values (Klimesch, 2012). This procedure allowed us to normalize individual scales and thus perform group analysis. So, the Alpha-OE ratio was analyzed in order to investigate its behavior along the DROW condition with respect to the other driving tasks. In fact, taking into consideration scientific literature related to the alpha spindles phenomena related to drowsy episodic events (Wang et al., 2020; Cui et al., 2021), as well as the neurophysiological results highlighting the overall higher alpha activity during the DROW task (Figure 4), we expected that the distribution of the alpha-OE ratio should show higher median values (i.e., the DROW condition caused an overall high alpha synchronization) with a large number of values higher than the median (i.e., alpha spindles should cause “peaks of alpha synchronization” along the task). Actually, the analysis of alpha-OE ratio distributions along all the driving tasks confirmed these working hypotheses, revealing how the distributions of this ratio were almost similar (i.e., without any significant difference) along the WUP, PERFO, and TAV tasks, while it was significantly higher along the DROW task, and even more, with a higher positive skewness. The latter result is the consequence of the presence of singular higher values, potentially linked to spindles events, and thus driving us towards the further development of the drowsiness index. Also, the similarity of WUP, PERFO, and TAV tasks is an important result of this study. In fact, these three driving tasks were different in terms of cognitive demand: with respect to WUP, PERFO included the specific request of increasing driving speed to improve performance. TAV even included a secondary task to accomplish while driving. Undoubtedly, these additional requests varied the cognitive demand by including new mental processes related to motor coordination, attention, vigilance, situation awareness, working memory, and even stress, thus potentially modifying the underlined neurophysiological activity (Alizadeh and Dehzangi, 2016; Borghini et al., 2017, 2020; Protzak and Gramann, 2018). Therefore, the result proved the robustness of such an index towards psychological phenomena other than drowsiness.

### Reliability and Novelty of the MDrow Index

Once validated the rationale of the drowsiness index from a physiological point of view, i.e., the possibility of detecting alpha synchronization events over the parietal sites as a potential cue of alpha spindles, our study went ahead by looking for a method for recognizing such “peaks of synchronization”. At this point, we hypothesized that spurious synchronization phenomena could happen also while driving with a “normal state”, therefore there was the need of establishing an individual threshold that, if overcome, would indicate a potential “altered state” (due to drowsiness insurgence). The WUP task, being not different from PERFO and TAV in terms of data distribution (Figure 5), was employed as a sort of ‘reference task’ to estimate the individual thresholds of “normal state”, therefore the Alpha-OE ratio peaks overpassing such a threshold were detected for each subject and for each task (Figure 6). Firstly, the analysis of the Alpha-OE ratio peaks’ rate, duration, and amplitude demonstrated that actually the mental drowsiness while driving occurred as a punctual phenomenon during the DROW condition. In fact, the peaks rate was significantly higher during such a condition compared to the others (all *p* < 0.05). Moreover, the peaks amplitude and duration were significantly higher during the DROW condition (all *p* < 0.05). This evidence confirms that during the DROW condition the alpha spindles were significantly stronger and longer than in the other conditions. To this regard, coherently with the definition of EEG GFP, this parameter takes into account and amplifies all the EEG synchronizations of Alpha rhythms within the time windows in which it is calculated. Therefore, the more Alpha peaks occur, the higher the EEG GFP in this band. Consequently, the EEG GFP in Alpha band relevantly increases in terms of amplitude and time width when the alpha spindles occur. The three Alpha-OE ratio parameters were then combined in a synthetic index, the MDrow, computed as the convolution, which is computationally equivalent to a moving average, of the Alpha-OE ratio index with a rectangular window. The statistical analysis performed on the MDrow index showed a significant increase during the DROW condition (all *p* < 0.05). More importantly, the non-zero percentage of MDrow index was above 50% during the DROW condition, while it was lower than 12% during the WUP and PERFO conditions (Figure 8).

The repeated measure correlation analysis showed that the MDrow index was significantly correlated with the EBR’ during the DROW condition. The analysis of the EBR’ confirmed that participants’ eye blinked significantly more frequently during the DROW condition compared to others (all *p* < 0.05). However, the Pearson’s correlations between the MDrow index and the EBR’ per each participant were varying between a large range, and for some of them, they were negative. This can be explained by hypothesizing that along the entire DROW condition factors other than the driving drowsiness impacted the EBR’ variations. It can be argued that in the first part of the DROW condition when the driving drowsiness was supposed to be less present compared to the final part of the condition, the participants’ eye movements were still detected as the result of other confounding factors (e.g., driving effort requested to the participants, variation of brightness, mind wandering, unconditional reflexes, etc.) undermining the reliability of the index (i.e., EBR) itself (Gonçalves and Bengler, 2015; Bajaj et al., 2021). Such a hypothesis is supported by the time dynamics of the MDrow along the experimental conditions (Figure 9). During the final part of the DROW condition, the MDrow index relevantly increases, while it is almost constantly zero along the other conditions. These considerations, taken together with the statistical analysis performed on the ECG parameters, which showed a significant difference in the HR and HRV only between the DROW and PERFO conditions, are highly supporting the hypothesis that other physiological measures based on ocular blinking and heart activity are able to discriminate prolonged states, but are less sensitives towards episodic shorts events such as alpha spindles. In other words, the EEG MDrow index has a higher time resolution enabling the detection of such events.

### Limitations and Future Improvements

Despite the promising results, there are some limitations and future improvements to be discussed. The experimental protocol was designed to induce mental drowsiness while performing the last condition (DROW), since the participants were asked to perform a simulated driving task for about 10 min on a monotonous and very low-speed track. It can be argued that the proposed MDrow index would be exclusively sensible to specific driving conditions. However, the present study aimed at validating the reliability of a synthetic EEG-based index in detecting driving drowsiness. A future step will consist in the application of such an approach in a more realistic context. A potential limitation of the presented work is the need of collecting the EEG signal from all the parietal channels, which could result in a high grade of invasiveness of the proposed methodology. The current implementation of the MDrow index is a starting point. Future work will consist of evaluating the driving drowsiness through an MDrow index derived from a simplified EEG system configuration, with the objective of reducing the required number of EEG channela. In this context, the MDrow index derived from only the frontal EEG channel will be tested. Collecting the EEG signal from such a region would imply a lower grade of invasiveness, paving the way to apply the proposed approach also in realistic driving environments. In addition, recent technological progress in EEG sensors and devices will bring to less and less invasive systems (Di Flumeri et al., 2019a), promoting the application of EEG-based monitoring techniques in daily life.

## Conclusion

The present study, through a simulated driving protocol, aimed at developing and validating an innovative and synthetic EEG-based index to evaluate driving drowsiness. The results confirmed the reliability of the proposed MDrow index in detecting mental drowsiness while driving. Furthermore, the MDrow index resulted to be more reliable and more sensitive in detecting the driving drowsiness episodes compared to the eye movement and ECG-derived parameters. The MDrow index was defined as the convolution between a parameter derived from the EEG GFP in the Alpha band and a rectangular window and, therefore, is compatible with an online implementation for the mental drowsiness evaluation while driving. In conclusion, the present work, besides the obtained results, paves the way to deploy the neurophysiological evaluation of the driving drowsiness in a realistic environment in order to positively contribute to road traffic safety.

## Data Availability Statement

The raw data supporting the conclusions of this article will be made available by the authors, without undue reservation. Please contact the corresponding author for any request.

## Ethics Statement

The studies involving human participants were reviewed and approved by Sapienza University of Rome. The patients/participants provided their written informed consent to participate in this study.

## Author Contributions

GB, GDF and VR: conceptualization, methodology, investigation, and data curation. GDF and VR: formal analysis and writing—original draft preparation. GB, PA, NS, and FB: resources. GDF, VR, AV, AG, NS, HZ, GDF, and WK: writing—review and editing. GB and FB: supervision. GDF, GB, and FB: funding acquisition. All authors contributed to the article and approved the submitted version.

## Funding

This research was co-funded by European Commission through the Horizon 2020 Framework Programme projects “FITDRIVE: Monitoring devices for overall FITness of Drivers” (GA n. 953432); “WORKINGAGE: Smart Working environments for all Ages” (GA n. 826232); “SIMUSAFE: Simulator Of Behavioral Aspects For Safer Transport” (GA n. 723386); “SAFEMODE: Strengthening synergies between Aviation and maritime in the area of human Factors towards achieving more Efficient and resilient MODE of transportation” (GA n. 814961); “MINDTOOTH: Wearable device to decode human mind by neurometrics for a new concept of smart interaction with the surrounding environment” (GA n. 950998); and H2020-SESAR-2019-2 project: Transparent artificial intelligence and automation to air traffic management systems, “ARTIMATION,” (GA n. 894238). The individual grants “BRAIN2GETHER” (BE-FOR-ERC) and “NEUROSIM” (Avvio alla ricerca, 2020), recognized by Sapienza University of Rome to GDF are also acknowledged.

## Conflict of Interest

GDF, VR, AG, AV, PA, NS, FB, and GB were employed by BrainSigns srl. The remaining authors declare that the research was conducted in the absence of any commercial or financial relationships that could be construed as a potential conflict of interest.

## Publisher’s Note

All claims expressed in this article are solely those of the authors and do not necessarily represent those of their affiliated organizations, or those of the publisher, the editors and the reviewers. Any product that may be evaluated in this article, or claim that may be made by its manufacturer, is not guaranteed or endorsed by the publisher.
